# Modulation of expression of heat shock proteins and apoptosis by *Flueggea leucopyrus* (Willd) decoction in three breast cancer phenotypes

**DOI:** 10.1186/s12906-015-0927-6

**Published:** 2015-11-09

**Authors:** Anuka S. Mendis, Ira Thabrew, Sameera R. Samarakoon, Kamani H. Tennekoon

**Affiliations:** Institute of Biochemistry, Molecular Biology and Biotechnology, University of Colombo, 90, Cumaratunga Munidasa Mawatha, Colombo, 03 Sri Lanka

**Keywords:** *Flueggea leucopyrus* (Willd.), Breast cancer phenotype cells, Heat Shock Proteins, Apoptosis, Anti-cancer

## Abstract

**Background:**

During the past few years, there has been an increasing interest among the Traditional and Folk medical practitioners of Sri Lanka in the use of a decoction prepared from *Flueggea leucopyrus* (Willd.) for treating various cancers including breast cancer. In the present study, the cytotoxicity of this decoction and its effects on Heat Shock Protein (HSP) expression and apoptosis were compared in three breast cancer phenotypes, to scientifically evaluate if a decoction prepared from *F. leucopyrus* (Willd.) is useful for the treatment of breast cancer.

**Methods:**

Cytotoxic potential of the *F. leucopyrus* decoction was determined by evaluating its effects in MCF-7, MDA-MB-231 and SKBR-3 breast cancer cell lines, and MCF-10A (non-cancerous) breast cell line, by use of the Sulphorhodamine (SRB) assay. The effect of the decoction on HSP gene expression in the above cells was evaluated by (a) Real time reverse transcription PCR (RT-PCR) and (b) Immunofluorescence analysis of HSP protein expression. Effects of the decoction on apoptosis were evaluated by (a) fluorescent microscopic examination of apoptosis related morphological changes and (b) DNA fragmentation (c) Caspase 3/7 assay.

**Results:**

*F. leucopyrus* decoction can mediate significant cytotoxic effects in all three breast cancer cells phenotypes (IC_50_ values: 27.89, 99.43, 121.43 μg/mL at 24 h post incubation periods, for MCF-7, MDA-MB-231, SKBR-3 respectively) with little effect in the non-cancerous breast cell line MCF-10A (IC_50_: 570.4 μg/mL). Significant (*P <0.05) inhibitions of HSP 90 and HSP 70 expression were mediated by the decoction in MCF-7 and MDA-MB-231, with little effect in the SKBR-3 cells. Clear apoptotic morphological changes on Acridine orange/Ethidium bromide staining and DNA fragmentation were observed in all three breast cancer cell lines. Caspase 3/7 were significantly (*P <0.05) activated only in MDA-MB-231 and SKBR-3 cells indicating caspase dependent apoptosis in these cells and caspase independent apoptosis in MCF-7 cells.

**Conclusions:**

Modulation of HSP 90 and HSP 70 expressions is a possible mechanism by which the decoction of *F. leucopyrus* mediates cytotoxic effects MCF-7 and MDA-MB-231 cells. This effect appears to correlate with enhanced apoptosis in these cells. In SKBR-3 cells, mechanisms other than HSP inhibition may be utilized to a greater extent by the decoction to mediate the observed cytotoxic effects. Overall findings suggest that the decoction has the potential to be exploited further for effective treatment of breast cancer.

## Background

During the past few years, much attention has been focused on exploiting the cytostatic and cytotoxic effects of phytochemicals to discover novel and effective treatment modalities in different types of human breast cancer [1]. Results of such investigations have led to the identification of a number of phytochemicals with potential to be beneficial in the management of breast cancer [2–6]. *Flueggea leucopyrus* (Willd.) also known as, Bushweed, is a medicinal plant (family: Phyllanthaceae) that grows commonly in certain regions of India, Myanmar, Pakistan and Sri Lanka [7, 8]. The leaves of this plant are traditionally used to prepare an extract or paste that is utilized as an alternative to commonly used antibiotics to destroy maggots in sores [7], to treat myiasis and promote wound healing [8], and for treatment of Otitis media [9]. In Sri Lanka, many indigenous medical practitioners have been using a decoction prepared from this plant for the treatment of a variety of cancers despite lack of any data from scientifically controlled trials to validate such claims. However, recent research conducted in our laboratory has provided experimental evidence to confirm that a decoction prepared from the aerial parts of this plant can exert a dose dependent cytotoxicity to AN3CA cells (derived from a hormone and chemotherapy resistant endometrial cancer), thus providing experimental support for the traditional use of *F. leucopyrus* for cancer therapy. Apoptosis and antioxidant activity have been demonstrated to be possible mechanisms through which such an effect is mediated [10]. A recent study done by Soysa et al. [11] has also demonstrated that aqueous extract of *F. Leucopyrus* leaves can inhibit the proliferation and induce apoptosis in Hep2 cells. Breast cancer is one of the most common female cancers and cause of cancer related deaths for women in most developed countries [12, 13]. In Sri Lanka, breast cancer has also become the most prevalent form of cancer affecting women [14, 15]. According to current views, breast cancer can be considered to be a collection of diseases characterized by malignant cells of different molecular phenotypes. These tumour subtypes are primarily recognized by expression of three cellular receptors, (a) estrogen receptor (ER, HGNC gene symbol ESR1), (b) progesterone receptor (PR, HGNC gene symbol PGR) and (c) the epidermal growth factor receptor family member Her2/Neu (HGNC gene symbol ERBB2). Because variations in the type of response expected from a particular therapeutic agent in breast cancer patients may occur due to variations in the phenotypic subtypes, increased attention is currently being given to strategies targeted to breast cancers based upon molecular subtypes [16–20]. However, despite the widespread clinical efficacy of these treatments, they are ineffective as therapy for receptor negative disease, and even among those patients that do initially respond, intrinsic or acquired therapeutic resistance remains a major obstacle to an effective cure indicating the need to develop novel therapeutic agents with increased selectivity and efficacy [21]. Although apoptosis is generally considered to be the most powerful defence mechanism against cancer [22], currently, there is increasing interest worldwide on the role played by Heat Shock Proteins (HSPs) in cancer development and the ability of anti-carcinogenic natural compounds and other anticancer drugs to inhibit their action. HSPs are often over expressed in many cancers, and HSP 70 and HSP 90 have been reported to be over expressed in different types of breast cancer cells [23–25]. Their expression often correlates with increased cell proliferation, lymph node metastasis, poor response to chemotherapy and poor survival [26]. HSPs have therefore surfaced as promising new targets for anti-cancer drug discovery. Therefore, an evaluation of the effects of the decoction prepared from *F. leucopyrus* on expression of HSPs in breast cancer cells would be very useful for value addition to these traditional therapies, as well as their further development into globally acceptable anticancer therapies for breast cancer. The present investigation was carried out with the main aims of (a) comparing the cytotoxicity of a *F. leucopyrus* decoction to different three breast cancer cell phenotypes, MCF-7 (ER positive / PR positive, Her2 negative), SKBR-3 (ER negative, PR negative, Her2 positive), and MDA-MB-231 (Triple negative), and a non-cancerous MCF-10A breast cell line, and (b) evaluating whether induction of apoptosis and modulation of HSPs (HSPs 70 and 90) are possible mechanisms by which the decoction mediates its anticancer effects in the above cell types.

## Method

### Chemicals and other reagents

Powdered Dulbecco’s Modified Eagle Medium and TRIzol reagent were purchased from Invitrogen Life Technologies (Carlsbad, CA, USA). MCF-7, MDA-MB-231, SKBR-3 and MCF-10A cell lines, Mc Coy’s 5A and L15 medium were purchased from American Type Culture Collections (ATCC; Manassas, VA). Sulphorhodamine (SRB), fetal bovine serum (FBS), streptomycin/penicillin, dimethyl sulfoxide (DMSO), agarose, and trypsin/EDTA were purchased from Sigma Aldrich Chemical Company (St. Louis, MO, USA). M-MLV reverse transcriptase system was purchased from Promega Cooperation, Madisons, U.S.A. PCR primers were purchased from Integrated DNA Technologies (IDT) U.S.A. Primary and secondary antibodies were purchased from Abcam (Cambridge, USA).

### Preparation of *F. leucopyrus* decoction

Aerial parts of the plants collected from Wewaldeniya, Western province, Sri Lanka and identity authenticated by the Botanist, National Herbarium, Department of National Botanic Gardens, Peradeniya, Sri Lanka, (voucher specimen NO: S 03 was deposited in the National Herbarium, Peradeniya, Sri Lanka) were dried at room temperature, and ground into powder using an electrical grinder. A decoction (aqueous extract) was prepared according to the method recommended traditionally for administration to cancer patient [10]. Sixty grams (60 g) of ground plant material was boiled gently with 1.6 L distilled water for approximately 3 h to reduce the volume to 200 mL. The extract was then filtered through a layer of muslin, filtrate centrifuged at 3000 g for 15 min to remove any debris, and the supernatant freeze dried and stored at −20 °C until required.

### Cell culture

The breast cancer cell lines MCF-7 (ER positive/ PR positive, Her2 negative), MDA-MB-231 (Triple negative), SKBR-3 (ER negative, PR negative, Her2 positive) and non-cancerous breast cell line MCF-10A were cultured according to instructions provided by ATCC and maintained at 37 °C in a 95 % air, 5 % CO_2_ atmosphere, and 95 % humidity.

### Evaluation of cytotoxicity by Sulphorhodamine (SRB) Assay

The SRB cytotoxicity assay was performed according to the method of Samarakoon et al. [27]. Cells (5 × 10^3^/well) were plated in 96 well culture plates and incubated for 24 h with the culture medium. At the end of this period, the medium in each well was removed by aspiration and cells incubated with fresh medium containing different doses (25, 50, 75, 100, μg/mL) of the decoction. Treated cells were incubated for a further 24 h. Cells were then fixed by gentle addition of ice-cold 50 % trichloroacetic acid solution (50 μL) to the medium in each well overlaying the cells. The plates were then incubated for 60 min at 4 °C. Wells were rinsed five times with tap water and then cells were stained with 0.4 % SRB solution (100 μL stain/well) for 15 min at room temperature. SRB solution was then poured off and unbound dye removed by washing five times with 1 % acetic acid solution and the plates left to air dry. The bound SRB dye was then solubilised by adding unbuffered Tris-base solution (200 μL/well), and plates were placed on a plate shaker for 1 h at room temperature. Plates were then read at OD 540 nm, using a microplate reader (Synergy HT Micro Plate Reader, BIO-TEK INSTRUMENTS, USA) and the results expressed as a percentage cell viability (mean of control group – mean of treated group/control group × 100 %). All the experiments were carried out in triplicate. Paclitaxel (T 7402-Sigma) was used as the positive control. Negative controls received only the medium and DMSO.

### Effects of the *F. leucopyrus* decoction on expression of Heat Shock Proteins (90 &70): Real time PCR

MCF-7, MDA-MB-231, SKBR-3 breast cancer cells cultured for 24 h were incubated for 24 h with fresh medium containing different concentrations (10, 20 μg/ mL) of the decoction (test cells) or 1 % DMSO (control cells). To evaluate expression of HSP 70 and 90 [28], concentrations below the IC_50_value (IC_50_ indicates 50 % of the cells dead [29]) of the decoction were selected. Each assay was carried out in three independent experiment in triplicates. At the end of the incubation period (24 h) cells were harvested and used for total RNA extraction for reverse transcription PCR (RT- PCR). Total RNA was isolated from the cultured cells, using TRIzol reagent according to the method described by Samarakoon et al. [30]. Total RNA concentration in the final elutes was determined by using a spectrophotometer (UV-1700, pharmasprc, SHIMADZU, Japan). Each sample of isolated RNA was reverse transcribed by M-MLV reverse transcriptase system. Real-time PCR reactions were performed in 96-well plates in Stratagene Mx 3000p real time PCR machine. Each reaction contained 1 μL of cDNA template, 0.5 μL of forward and reverse primers (Primers for HSP90 F 5″- CGCTCCTGTCTTCTGGCTTC - 3″, R 5″ - TGGTATCATCAGCAGTAGGGTCA −3″ and HSP 70 F 5″ - CCATCATCAGCGGACTGTACC −3″, R 5″- CTGACCCAGACCCTCCCTT −3″ [31]), 2X MESA GREEN qPCR Master Mix Plus for SYBR Assay (12.5 μL) bringing the final reaction volume to 25 μL with PCR water. All reactions were carried out in duplicate for each cDNA sample. As a control for genomic DNA contamination, an equivalent amount of total RNA without reverse transcription was tested. A no-template control (NTC) was also included in each run for each gene. Finally, a dissociation curve was generated by increasing temperature starting from 65 to 95 °C to determine the accuracy and specificity of the reactions. The crossing cycle number (Ct) or the threshold cycle defined as the PCR cycle at which the fluorescent signal of the reporter dye crosses an arbitrarily placed threshold was automatically determined for each reaction by the Mx 3000p software [32]. Expression of HSP 90 and HSP 70 in the decoction treated breast cancer cells were determined by Real time PCR using a comparative threshold cycle method and the fold change in expression of each gene was normalized to a house keeping gene (GAPDH gene) [32].

### Immunofluorescence analysis and quantification of HSP 90 & HSP 70 expression

Cells (5 × 104) were grown on UV sterilized cell culture coverslips (Nunc 174950 Thermanox) in a 24 well plate and maintained for 24 h. Breast cancer cells were then exposed to different concentrations (10, 20, 40 μg/mL) of the decoction (control cells were kept free from the decoction) for 24 h. Cells were then rinsed with Phosphate buffer saline (PBS) twice. To fix the cells, ice cold acetone (400 μL) was added to each well and kept in 20 °C freezer for 20 min. Cells were again washed twice with PBS. Fixed cells were incubated with PBS containing 0.25 % Triton X-100 (PBST) and 1 % BSA (400 μL) for 30 min at room temperature to block unspecific binding of the antibodies. Cells were then incubated with 1:200 primary antibody (diluted in 1 % BSA in PBST) overnight at 4 °C. Solution was decanted and cells were washed with PBS five times (5 min for each time). Cells were then incubated with the secondary antibody conjugated with fluorescein isothiocyanate (FITC) at 1:200 dilutions for 1 h at room temperature in the dark. Unbound secondary antibody was decanted and cells washed three times with PBS (5 min each time). Coverslips were carefully removed from the wells and placed on glass slides. The cover slips were dried before mounting with Dako fluorescent mounting medium (Dako, USA). Samples were analysed under a fluorescence microscope (Olympus, BX51TRF, Japan) with 200 × objective at the relevant wavelengths. Images were recorded digitally using the LSM software (Zeiss, Germany). With each image capture, care was taken that the same parameters were used. Non-specific staining was not observed upon staining with secondary antibodies only.

Same procedure as described in immunofluorescence analysis was carried out, except it was carried out in a 96 well plate. To 10 thousand cells in each well 1/5 of the volume indicated above for each reagent was added. Quantification was carried out obtaining the fluorescence reading via the area scan of each well by the Synergy HT microplate reader.

### Detection of morphological changes related to apoptosis by fluorescent microscopy

MCF-7, MDA-MB-231 and SKBR-3 cells were grown on cover slips at a final concentration of 4 × 10^5^ cells/mL in 24 well culture plates for 24 h. The cells were then exposed to different concentrations of the decoction (10, 20 & 40 μg/mL) (test cells) or 0.1 % DMSO (control cells) respectively for 24 h. The cells were fixed by 4 % formaldehyde at room temperature and plated onto glass slides and subjected to apoptosis analysis by Acridine orange/Ethidium bromide (AO/EB) staining as described in Samarakoon et al. [31, 32]. Changes in the nuclei of cells were observed within 15 minutes after AO/EB staining under a fluorescence microscope (Olympus, BX51TRF, and Japan).

### DNA fragmentation analysis

DNA fragmentation was used to detect the characteristic ladder pattern of DNA fragmentation that occurs during apoptosis, as described by Samarakoon et al. [10] with slight modifications. Cells (2 × 10^5^ cells/mL) were exposed to the decoction for 24 h. Trypsinisation was carried out and the pellets were incubated for 60 min at 50 °C in 100 μl lysis buffer (100 mM Tris–HCl pH 8, 100 mM NaCl and 10 mM EDTA). Proteinase K (10 μl 20 mg/mL stock solution) was then added to the lysis mixture and further incubated for 30 min at 50 °C. RNase (3 μL from 10 mg/mL stock solution) was then added and the mixture incubated for 2 h at 50 °C. DNA was extracted with phenol chloroform-isoamyl alcohol, subjected to 2.0 % of agarose gel electrophoresis, stained with ethidium bromide and visualized under UV light using a gel-doc system (Quantum- ST4 1100/20 M).

### Caspase 3/7 activity

Effect on caspase 3/7 activity was determined in the three breast cancer cell lines. Cells were treated with the decoction for 24 h (10, 20, 40 μg/ml). Caspase activity was assessed using ApoTox-Glo™ triplex assay as per manufacture’s recommendations (Promega, G6321) and compared with untreated controls.

### Statistical analysis

Statistical analysis was carried out by using Prism 5.0 software (Graphpad Prism, San Diego, CA). The IC_50_ values were obtained through Non-linear regression analysis for SRB analysis. Effect of the decoction on HSP expression were analysed by using one way ANOVA with Dunnett’s multiple comparison test. Effect of the decoction on Caspase 3/7 activity were analysed by using one way ANOVA with Tukey: Compare all pairs of columns test.

## Results and discussion

### Effect on relative cell survival

**Fig. 1:**
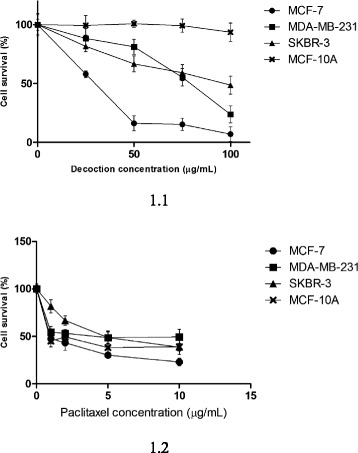
Cell survival (by SRB assay) of all three breast cancer cells (MCF-7, MDA- MB-231 & SKBR-3) and normal breast cell line (MCF-10A). **1.1** Treated with the *F. leucopyrus* decoction for 24 h. Data values are expressed as mean ± SD of eight replicates. **1.2** Treated with Paclitaxel for 24 h (Positive control). Data values are expressed as mean ± SD of eight replicates

In a previous investigation Samarakoon et al. [10] have demonstrated that a decoction prepared from the aerial parts of *F. leucopyrus* (Willd.) can mediate significant cytotoxicity to AN3CA cells (estrogen receptor negative endometrial carcinoma cells) which are sensitive to hormone therapy. In this study we report for the first time the cytotoxic effects of the decoction on three breast cancer phenotypes [MCF-7 (ER positive / PR positive, Her2 negative), SKBR-3 (ER negative, PR negative, Her2 positive), and MDA-MB-231 (Triple negative)]. Results of the present study demonstrate that *F. leucopyrus* decoction can exert significant cytotoxic effects in all three different phenotypes of breast cancer cells under test. Results also demonstrate that the decoction can exert selectively greater cytotoxicity to the breast cancer cell lines than the non-cancerous breast cells MCF-10A. Thus as apparent from Fig. 1, that summarizes results of the SRB assay, on incubation of the cells for 24 h with the decoction, a dose dependent inhibition of cell survival was observed with IC_50_ values of 27.89, 99.43, 121.43, 570.4 μg/mL at 24 post incubation periods, for MCF-7, MDA-MB-231, SKBR-3 and MCF-10A respectively. IC_50_ values of the positive control paclitaxel at 24 h were 1.44, 3.47, 4.9, 2.01 μg/mL for MCF-7, MDA-MB-231, SKBR-3 and MCF-10A respectively. Higher concentrations of the decoction were used as the decoction is composed of many compounds and some of them may interact with the most cytotoxic compound of the plant. However smaller doses of paclitaxel were used, since it is a pure compound with IC_50_ values much lower than that of the decoction. Paclitaxel is a known chemotherapy drug used to treat different cancers including ovarian, breast and non-small cell lung cancer. It interferes with normal microtubule growth of the cell by hyper stabilising its structure [33]. The cytotoxic and apoptotic effects of paclitaxel have been utilised as a positive control in a study done by Moongkarndi et al. [34] in Ovarian and breast cancer cell lines.

Among the three breast cancer cell phenotypes, the decoction (according to IC_50_ values obtained in the SRB assay) appeared to be selectively more cytotoxic to MCF-7 cells than the other two breast cancer cell types. Overall results suggest that *F. leucopyrus* is selectively more cytotoxic to the Her2 negative cell lines (MCF-7 and MDA-MB-231) than to Her2 positive cell line SKBR-3. Similar results have been reported for the natural compound Curcumin [5]. A recent study [35] has also shown that the highly invasive cell lines such as MDA-MB-231 were more sensitive to the cytotoxic effects of the natural compound Magnanol than the less invasive cells such as MCF-7 and SKBR-3. In contrast, Genistein (an isoflavone isolated from Soya) has been reported to exert stronger antiproliferative effects in Her2 positive breast cancer cell lines than in Her2 negative MCF-7 cells [36]. Such variations in sensitivity to the cytotoxic effects may be due to differences in the rates of absorption of the active compounds into the different breast cancer cell phenotypes as suggested in the Curcumin study by Altenberg et al. [5] or may be due to differences in the mechanisms utilized by these compounds to mediate anti-proliferative effects.

### Modulation of HSP 90 and HSP 70 expression in Her2 negative phenotypes and Her2 positive phenotype

Heat shock proteins are emerging as important molecules in cancer development and are considered to be key targets in cancer therapy. These molecular chaperones are over - expressed in many human cancers, including breast cancer [23–25, 36, 37] and enhance cancer cell proliferation, and protect tumours from therapies such as chemotherapeutic drugs and surgery, used in cancer management [26]. In breast cancer cells, over expression of HSP 90 and HSP 70 are reported to correlate with poor prognosis. Recent investigations have demonstrated that inhibition of breast cancer cell proliferation by certain plant based compounds such as Curcumin and the flavanoidal compounds quercetin and taxifolin [23], may in part be mediated via significant inhibition of HSP expression in these cells. In this study, statistically significant inhibition of HSP expression by the *F. leucopyrus* decoction was observed in only two of the breast cancer cell phenotypes (MCF-7, MDA-MB- 231) under investigation, but not in the SKBR-3 phenotype. This observation was apparent on evaluation of the HSP mRNA as well as HSP protein expressions in the cells by use of Real time PCR and immunofluorescence techniques respectively. Such a difference in HSP expression may be a reason for the observed differences in cytotoxicity mediated by the decoction in the MCF-7, MDA-MB-231, and SKBR-3 cells.

On evaluation of HSP 90 gene expression by RT- PCR (Fig. 2.1) in MCF-7 (Fig. 2.1a) and MDA-MB-231 (Fig. 2.1b) cells treated with either 10 μg/mL or 20 μg/mL of the decoction, significant (*P <0.05) reduction in HSP 90 gene expression was observed when compared to non-treated cells (One way Anova with Dunnett’s multiple comparison test). In SKBR-3 (Fig. 2.1c) cells no significant effects were observed in comparison to the controls. These results are supported by the qualitative (Fig. 2.2) immunofluorescence results. The intracellular localization of HSP 90 signal in MCF-7 (Fig. 2.2a) or MDA-MB-231 (Fig. 2.2b) cells were observed to be more intense in the cytoplasm than in the nucleus. On Visual observation, the intensity of signal in cell cytoplasm appeared to decrease with increasing concentration of the decoction, though the nuclear staining remained relatively unchanged. Quantitative analysis of immunofluorescence (Fig. 2.3) showed significant (*p <0.05) reduction in expression of HSP 90 gene of treated MCF-7 (Fig. 2.3a) or MDA-MB-231 (Fig. 2.3b) cells when compared to non-treated cells. Qualitative and quantitative immunofluorescence results of HSP 90 in SKBR-3 (Fig. 2.2c and 3c) cells did not show any significant reduction as the intensity of the signal were maintained with increasing concentration of the decoction.

**Fig. 2:**
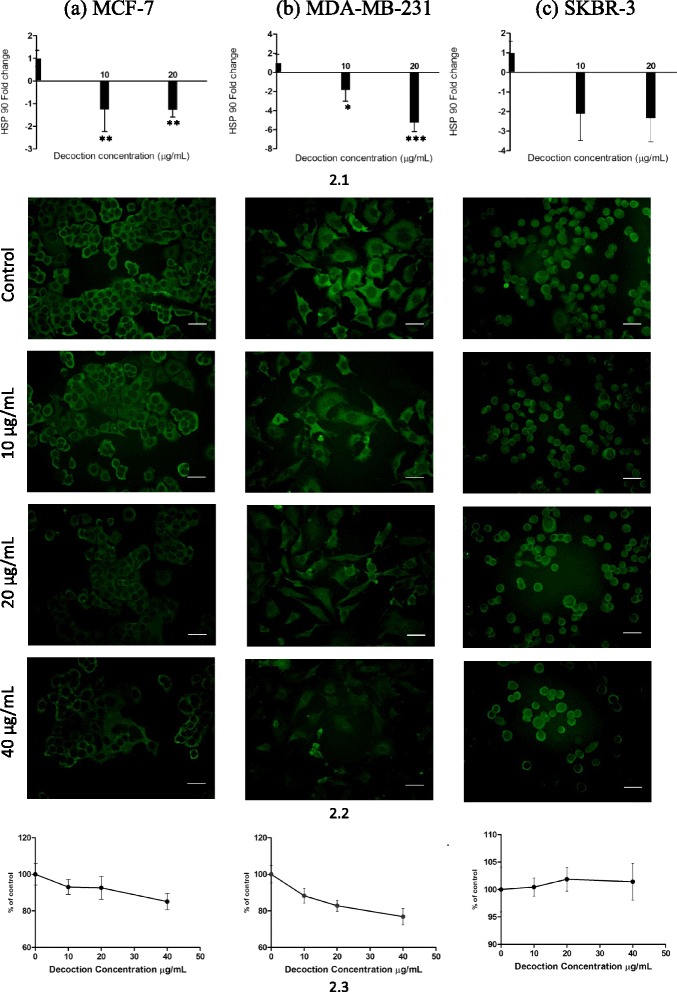
Effects of the *F. leucopyrus* decoction on HSP 90 expression in (*a*) MCF-7, (*b*) MDA-MB-231 & (*c*) SKBR-3 breast cancer cells. **2.1** Real time PCR results of HSP 90 gene expression. Data are representative of three independent experiments (mean ± SD of three replicates). **2.2** Qualitative Immunofluorescence results, Scale bar: 100 μm. **2.3** Quantitative Immunofluorescence results. Data are representative of five independent experiments (mean ± SD of five replicates)

**Fig. 3:**
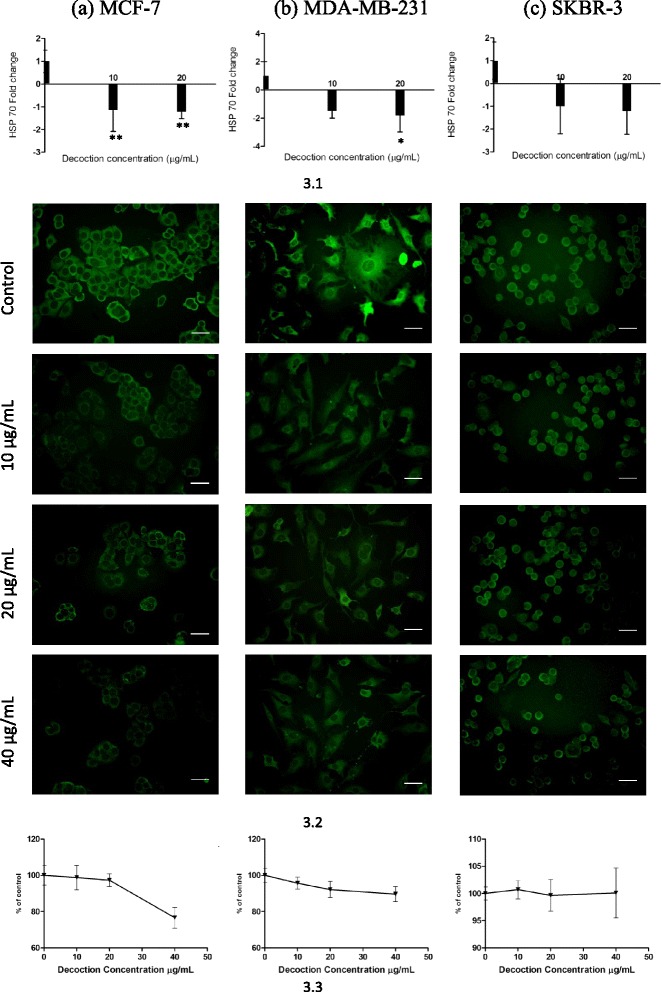
Effects of the *F. leucopyrus* decoction on HSP 70 expression in (*a*) MCF-7, (*b*) MDA-MB-231 & (*c*) SKBR-3 breast cancer cells. **3.1** Real time PCR results of HSP gene expression. Data are representative of three independent experiments (mean ± SD of three replicates). **3.2** Qualitative Immunofluorescence results, Scale bar: 100 μm. **3.3** Quantitative Immunofluorescence results. Data are representative of five independent experiments (mean ± SD of five replicates)

Similar results were observed with HSP 70 gene expression. In the RT- PCR evaluation of HSP 70 (Fig. 3.1) gene expression in MCF-7 (Fig. 3.1a) or MDA-MB-231(Fig. 3.1b) cells treated with either 10 μg/mL or 20 μg/mL significant (*P <0.05) reduction in HSP 70 gene expression was observed when compared to non-treated cells (One way Anova with Dunnett’s multiple comparison test). In SKBR-3 cells no significant effects in comparison to controls were observed in the expression of HSP 70 (Fig. 3.1c), even though a significant dose dependent cytotoxicity was mediated in these cells by the decoction, as demonstrated by results of the SRB assay. Visual observation of HSP 70 immunofluorescence (Fig. 3.2) supported the results obtained from the RT-PCR evaluation. The intracellular localization of HSP 70 signal in MCF-7 (Fig. 3.2a) or MDA-MB-231 (Fig. 3.2b) cells were also observed to be more intense in the cytoplasm than in the nucleus and the intensity of the signal in cell cytoplasm appeared to decrease with increasing concentration of the decoction, though the nuclear staining remained relatively unchanged. Quantitative analysis of immunofluorescence showed significant (*p <0.05) reduction in both MCF-7 (Fig. 3.3a) and MDA-MB-231 (Fig. 3.3b) cell lines. However no significant reduction was observed in the qualitative and quantitative immunofluorescence results of HSP 70 (Fig. 3.2c and 3c) in SKBR-3 cells.

**Fig. 4:**
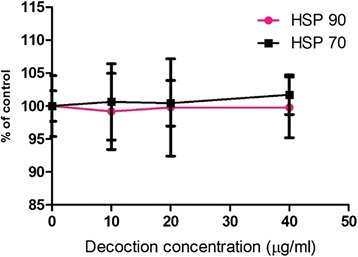
Effects of the decoction on HSP 70 and HSP 90 expression in MCF 10a cells treated with *F. leucopyrus* decoction for 24 h. Quantitative Immunofluorescence results of HSP 70 and HSP 90 gene expression in MCF 10a cells mediated by the different concentrations of the decoction, (as analyzed by One way ANOVA with Dunnett’s post test) Data values are expressed as mean ± S.D

In comparison to the quantitative immunofluorescence results of HSP 90 and HSP 70 expression in the three breast cancer cell line no significant effects compared to the controls were observed in in MCF 10a (Fig. 4) normal breast cell line treated with the different concentrations of decoction.

In contrast to results obtained in the cytotoxicity study, overall results of the HSP evaluation demonstrates that HSPs of the highly invasive MDA-MB-231 cells were more sensitive to the *F. leucopyrus* decoction than that of the poorly invasive MCF-7 and SKBR-3 cells. Thus, on RT-PCR evaluation of HSP mRNA expression, at a dose of 20 μg/mL of decoction, inhibition of HSP 90 in MDA-MB-231 was significantly greater (about 2.5 fold) than in MCF-7 cells although HSP70 expression was comparable in the two phenotypes. During the past decade, HSP 90 has emerged as an exciting target for cancer therapy because it has been shown to be involved in maintaining the conformation, stability, activity and cellular localization of several key oncogenic client proteins such as ERBB2, C-RAF, CDK4, AKT/PKB, steroid hormone receptors, mutant p53, HIF-1α and telomerase hTERT. It has been demonstrated to be linked to all six hall marks of cancer described by Hanahan and Weinberg [38] and modulation of this single drug target is considered to be effective in simultaneously inhibiting all the multiple signalling pathways and biological processes that have been implicated in the development of the malignant phenotype [38-40]. It is therefore interesting to find from the overall results of the RT-PCR and immunofluorescence evaluations carried out in the present investigation, that HSP 90 of the highly invasive MDA- MB-231 cells was more sensitive to the *F. leucopyrus* decoction than that in the poorly invasive MCF-7 cells, although effects of the decoction on HSP 70 expressions were comparable in the two phenotypes MDA-MB-231 and MCF-7.

### Effects of the decoction on apoptosis in the three breast cancer phenotypes

Apoptosis is a highly regulated process that is well characterized by distinct morphological and physiological changes in the cell. The morphological changes includes nuclear condensation, cell shrinkage and membrane blebbing, whereas the physiological changes are fragmentation of nuclear DNA due to activation of specific endonucleases cleaving nuclear DNA into 80–200 oligonucleosome fragments [41]. These apoptotic changes are mainly mediated by a group of intracellular proteases called caspases. There are two caspase activating cascades that regulate apoptosis they are the intrinsic and extrinsic pathway [42]. Apoptosis is resistant to high expression of HSP. Thus a decrease in HSP expression would be expected to result in increased apoptosis in a cell [43].

**Fig. 5:**
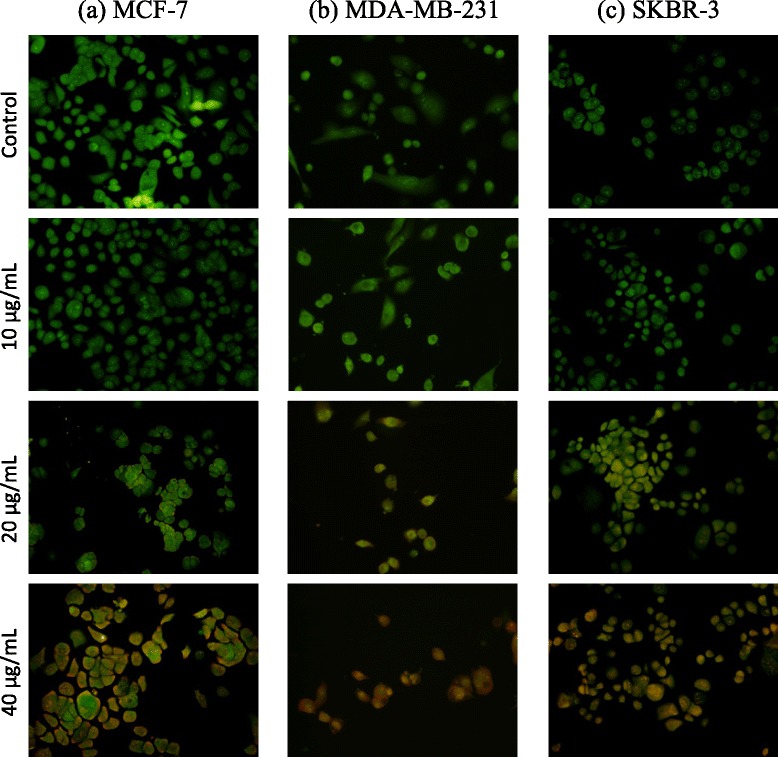
Effects of the *F. leucopyrus* decoction on apoptosis in the three breast cancer cell phenotypes. Fluorescent microscopic observations of morphological changes in **a** MCF-7, **b** MDA-MB-231 or **c** SKBR-3 cells (magnification 200×), following the treatment with different concentrations of the decoction, by AO/EB staining

Fluorescent microscopic observations of AO/EB stained (Fig. 5) in MCF-7 (Fig. 5a), MDA- MB-231(Fig. 5b) and SKBR-3 (Fig. 5c) cells, following the treatment with different concentrations of the decoction showed that the nuclei of viable cells stained uniformly green by acridine orange, while those of apoptotic cells exhibited yellow to orange coloration, depending on the degree of loss of membrane integrity, due to co-staining with ethidium bromide. In this experiment, yellow staining represented early apoptotic cells, while reddish orange staining represented late apoptotic cells [10]. A dose-dependent increase in induction of apoptosis was observed in the three breast cancer cells treated with the different doses of *F. leucopyrus* decoction for 24 h, as indicated by alterations in cell staining (described above).

**Fig. 6:**
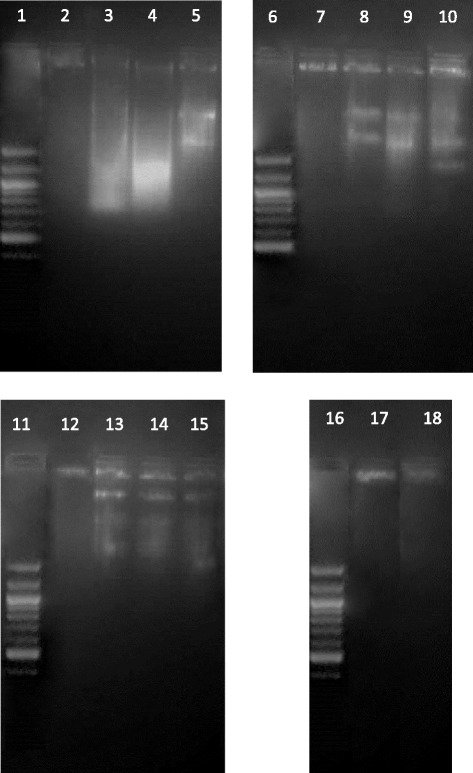
DNA fragmentations in MCF-7, MDA-MB-231 or SKBR-3 breast cancer cells treated with *F. leucopyrus* decoction. Lane 1- 100 bp ladder, Lane 2- MCF-7 Control, Lane 3- MCF-7 Paclitaxel 5 μg/mL, Lane 4-MCF-7 Decoction 40 μg/mL, Lane 5-MCF-7 Decoction 100 μg/mL Lane 6- 100 bp ladder, Lane 7-MDA-MB-231 Control, Lane 8- MDA-MB-231 Paclitaxel 5 μg/mL, Lane 9- MDA-MB-231 Decoction 40 μg/mL, Lane 10- MDA-MB-231 Decoction 100 μg/mL, Lane 11- 100 bp ladder, Lane 12- SKBR-3 Control, Lane 13- SKBR-3 Paclitaxel 5 μg/mL, Lane 14- SKBR-3 Decoction 40 μg/mL, Lane 15- SKBR-3 Decoction 100 μg/mL, Lane 16- 100 bp ladder, Lane 17- MCF 10A Control, Lane 18- MCF10A Decoction 100 μg/mL

DNA fragmentation was observed in all three breast cancer cells exposed to the decoction for 24 h. Untreated control cells showed no evidence of DNA fragmentation while the smeared laddering pattern was observed in cells treated with 40 and 100 μg/mL of the decoction for 24 h. DNA fragmentation was done at higher doses as this event occurs at the execution phase of apoptosis [44]. All three breast cancer cell lines treated with positive control paclitaxel at 5 μg/mL showed DNA fragmentation. MCF 10A was also treated with 100 μg/mL of the decoction and did not show any laddering pattern (Fig. 6).

To confirm apoptosis quantitatively, caspase 3/7 assay was performed as described in the Methods section. As evident from Fig. 7.1, no significant activity was detected in MCF-7 cells (*P <0.05). However in MDA-MB-231 cells a statistically significant (*P <0.05) dose dependent increase in caspase activity was observed at doses <20 μg/ml (Fig. 7.2) and in SKBR-3 cells at doses >20 μg/ml (Fig. 7.3).

**Fig. 7:**
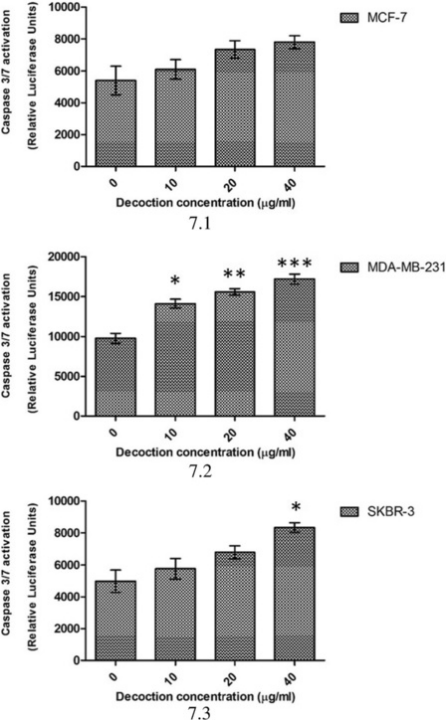
Activation of Caspase 3/7 in breast cancer cells 24 h after treatment with the decoction. ***p <0.05. **7.1** MCF-7, **7.2** MDA-MB-231 cells, **7.3** SKBR-3 cells. Data are representative of three independent experiments (mean ± SD of three replicates)

Caspase independent pathway (intrinsic pathway or mitochondrial pathway) is activated in response to death stimuli such as DNA damage, chemotherapeutic agents, and UV radiation [41]. Upon activation, results in the release of cytochrome c (a complex of the mitochondrial electron transport chain) into the cytosol from the mitochondria. Cytochrome c function is altered and binds to a procaspase activating adapter protein known as apoptotic protease activating factor 1 (Apaf1) forming a proteasome complex which recruits procaspase 9 along with dATP that cleaves and activates the precursor to caspase 9. Downstream activation of the procaspases continues inducing apoptosis [41, 45, 46].

HSP 70 or 90 binds to the Apaf-1, thereby inhibiting caspases activation and apoptotic cell death. HSP70 has also been shown to block caspase independent cell death through its association with apoptosis inducing factor (AIF) [47]. In this present study there was no significant increase in expression of Caspase 3/7 mediated in MCF-7 cells by the decoction as caspase 3 is not expressed in MCF-7 according to publish reports [48]. However the decoction significantly inhibited HSP 70 and 90 in MCF −7 cells, hence it can be concluded that in these cells apoptosis can occur via a caspase independent pathway.

In the present investigation it was observed that exposure of the decoction to MDA-MB-231 cells significantly increases Caspase 3/7 expression. Caspase dependent pathway otherwise known as the extrinsic pathway is another pathway that occurs in apoptosis [41]. Extrinsic pathway is triggered when transmembrane receptors such as fibroblast antigen signalling receptor (FAS), tumor necrosis factor (TNF), TRAIL and death receptor (DR36) ligates with complementary ligands such as FasL and TNFα. On activation, each receptor can form a death inducing signalling complex (DISC) by recruiting the adaptor Fas-associated death domain (FADD) and apical procaspase 8 and 10. Activated caspase 8 and 10 cleaves and activates caspase 3 and 7 resulting in apoptosis [41, 45, 46]. Many elements of this pathway are regulated by the activities of HSP 70 and 90 to help maintain cellular survival following death receptor ligation. HSP 70 suppresses apoptosis by binding to and inhibiting TRAIL and TNF-induced apoptosis [49]. HSP 90 also regulates TNF-mediated cellular survival which has been linked to their ability to regulate the stability and activity of a number of components of the NF-κB activation pathway [49]. HSP 70 and 90 were inhibited by the decoction and there was an increased activity of caspase 3/7 suggesting apoptosis in MDA-MB-231 cells occurs via the extrinsic pathway.

Hsp90 is a homodimer, and comprises three domains. The N-terminal domain has an ATP-binding site, and natural products such as geldanamycin and radicicol binds to this domain. Co-chaperones and client proteins binds to the highly charged middle domain. ATP usually binds to the C-terminal nucleotide binding pocket but this domain has an affinity to cisplatin, novobiocin, epilgallocatechin-3-gallate (EGCG) and taxol [50]. A study done by Solit et al. [51] reported that the combination of HSP 90 inhibitor, 17-allylamino-17-demethoxygeldanamycin (geldanamycin derivative) and taxol can supress AKT activity and subsequently sensitize tumor cells to proapoptotic stimuli.

From the overall results obtained from MCF-7 and MDA-MB-231, inhibition of HSP’s along with increase in apoptotic activity may be induced by a single bioactive compound or through a synergistic effects of different compounds present in the decoction. Anticancer effect has been reported in HepG2 cells by synergistic effects among constituents in an ethanol-water crude extract of *Polyalthia evecta* (*P. evecta*) [52]. However results contradicting to those obtained from the MCF-7 and MDA-MB-231 cells was observed in SKBR-3 cell line. Although Her 2 amplified breast cancer cell lines have high HSP 90 protein levels, as it constitutively activates heat-shock factor 1 (HSF1) [53], inhibition of HSP 90 was not statistically significant when compared to the other breast cancer cell lines. Furthermore caspase 3/7 was activated when treatment with the decoction despite not having any significant inhibitory effect on the expression of HSP 70 and 90. The reason behind this is unclear, but a study carried out by Massy 2010 indicates that VER-155008 (ATP-derivative inhibitor of HSP70) inhibited the proliferation of human breast cancer cells but did not enable the apoptotic potential of a small molecule Hsp90 inhibitor in MDA-MB-468 cells [54].

Natural anticancer compounds have been reported to mediate their actions via several mechanisms such as NFkβ inhibition, apoptosis induction, and effects on microfilament aggregation, antioxidant activity, proteasome inhibition and HSP inhibition [55]. However, it is not clear which of them contributes mainly to the anticancer effects mediated by these compounds. It is possible that in SKBR-3 cells, mechanisms other than HSP inhibition maybe utilized to a greater extent by the *F. leucopyrus* decoction to mediate the observed cytotoxic effects. Development of effective anti-cancer drugs has come to a halt as there is lack of phenotype specific anti-cancer agents [56]. HSP inhibitor such as colchicine, geldanamycin (GA) are such drugs whose clinical development was restricted by their toxicity despite their effectiveness at the preclinical stage [51, 56, 57]. Therefore discovering new compounds that are phenotype specific and which impose less cytotoxicity to normal cells is crucial for cancer therapy [58]. *F. leucopyrus* decoction is a promising new candidate as its cytotoxicity is significant to breast cancer cell lines and imposes minimalistic effect on normal breast cell line. Further studies are necessary in the future to identify the bioactive compounds mediating the cytotoxic and other related effects in order to determine whether a single compound or a mixture of compounds in the decoction is responsible for the observed effects.

## Conclusions

*F. leucopyrus* decoction is significantly cytotoxic to all three breast cancer cell phenotypes used in this study (MCF-7, MDA-MB-231 and SKBR-3) with greater cytotoxicity being exerted in Her2 negative phenotypes MDA-MB-231 and MCF-7 than in the Her2 positive phenotype SKBR-3. The decoction also exhibits selective cytotoxicity to the breast cancer cells in comparison with the non-cancerous breast cell line MCF-10A. These results help to rationalize the ethnopharmacological claims regarding presence of anticancer properties in *F. leucopyrus*. Inhibition of heat shock protein expression (HSP 70 and HSP 90) and enhanced apoptotic activity as observed in the results of this study appears to be one of the mechanisms utilized by the decoction to mediate its cytotoxic effects in the Her2 negative cell lines. In the Her2 positive SKBR-3 cells, mechanisms other than HSP inhibition appear to be utilized to a greater extent by the *F. leucopyrus* decoction to mediate cytotoxic effects. Overall findings of the present study suggest that the *F. leucopyrus* decoction has the potential to be exploited further for effective treatment of breast cancer, particularly the Her2 negative phenotypes. Confirmation of the in vitro results obtained needs to be carried out in the future by use of appropriate in vivo models.

## Abbreviations

AO/EB: acridine orange/ethidium bromide
FITC: fluorescein isothiocyanate
HSP: heat shock proteins
SRB: sulphorhodamine
